# Anxiety and depression as aspects of influence on the quality of life in patients with lymphoproliferative diseases

**DOI:** 10.1192/j.eurpsy.2025.744

**Published:** 2025-08-26

**Authors:** S. V. Kuzmina

**Affiliations:** Psychiatry and medical psychology, Kazan State Medical University, Kazan, Russian Federation

## Abstract

**Introduction:**

The high prevalence of anxiety and depression among patients with cancer has an impact on a decrease in quality of life. there are practically no studies among people with lymphoproliferative diseases, which determines the relevance of our study.

Purpose: to assess the prevalence of anxiety-affective spectrum disorders and their impact on the quality of life and adaptability in patients with non-Hodgkin lymphoma (NHL), Hodgkin lymphoma (HL) and a group, including malignant neoplasm of the body of the stomach and T- and B- cellular lymphoma.

**Objectives:**

95 patients (women - 52, men - 44) diagnosed with NHL, HL, others were examined using a continuous sampling method.

**Methods:**

Clinical interviews; the author’s questionnaire; HADS, EQ-5D, EQ-VAS, PSM-25. STATTECH were used to process the data.

**Results:**

The average age was 52.8±8.3 years (18 to 86). 3 groups: 1-non–Hodgkin’s lymphoma, 2-Hodgkin’s lymphoma, 3- malignant neoplasm of the stomach body and T- and B-cell lymphoma. In gr-s 1 and 2 (90% and 85%), no symptoms of anxiety were detected. In gr 3, clinically pronounced anxiety levels were uniformly detected in 50%. Depressive symptoms a predominantly absence in all groups (1 - 95%; 2 - 86% and 3 - 75%) p>0.05. The assessment of quality of life: in gr 1 and 2, most of the patients noted that they experience some difficulties in the final indicator (55% and 61%), pronounced problems prevail in gr 3 (50%). The mobility of the majority in all groups does not suffer or suffers to a minor extent, 80% of all noted the absence of difficulties in self-care. “daily activity” were a statistically significant - 64% maintained it at a high level (p=0.006); Pain/discomfort is experienced by more than half of the respondents to a moderate degree 55% (p=0.003). In gr 1 and 3, the average of EQ-VAS determined at a level of more than 70 (the arithmetic mean 78 and 73), which indicates a more favorable sense of self. In gr 2 the average was determined at 68, also characterizes a high level of assessment of their general condition, in all patients with CLL, according to PSM 25, the stress level was determined to be low, indicates a state of psychological adaptation to workloads in 100%. In the gr 1, 12 people (17%) were identified with an average level of stress and reduced adaptation and need the rest for this contingent.

**Conclusions:**

our pilot study showed that patients with lymphoproliferative diseases are characterized by a slight decrease in their quality of life, a high level of life satisfaction, good stress resistance to workloads. The main symptoms (reliably expressed in 55%), are moderate severity of pain and discomfort. The obtained data make it possible to emphasize the heterogeneity of the prevalence and severity of anxiety-affective symptoms in patients with various types of oncological diseases.

**Disclosure of Interest:**

None Declared

